# Re-examining the Diagnostic Criteria for Wilson’s Disease: A Case Report and Literature Review

**DOI:** 10.7759/cureus.99271

**Published:** 2025-12-15

**Authors:** Lena Hummel, Christopher Carr, Simone Biow, Karen Asher, Theodor Sauer, Marlowe Maylin

**Affiliations:** 1 Department of Ophthalmology, University of Texas Medical Branch at Galveston, Galveston, USA; 2 Department of Neurosurgery, Medical College of Georgia at Augusta University, Augusta, USA; 3 Department of General Surgery, Houston Methodist Hospital, Houston, USA; 4 Ophthalmology, Ophthalmic Consultants of Boston, Boston, USA; 5 Department of Internal Medicine, Tulane University Medical Center, New Orleans, USA

**Keywords:** ceruloplasmin, copper, diagnosis, hepatolenticular degeneration, wilson’s disease

## Abstract

Wilson’s disease (WD) is a heterogeneous genetic disorder for which diagnosis is challenging. We present the case of a 54-year-old woman with a complicated medical history, including hepatitis C, cirrhosis, hepatic encephalopathy, and extensive psychiatric disease, who was transferred to our hospital for management of a spinal epidural abscess. Further findings suggested undiagnosed WD, including bilateral rings around Descemet’s membrane and modestly low ceruloplasmin but normal urinary copper excretion. Many algorithms have been proposed for Wilson disease diagnosis, including clinical, laboratory, imaging, and genetic findings; however, no single test is diagnostic. The European Association for the Study of the Liver (EASL) guidelines are the most commonly employed algorithm. Updated 2022 guidelines by both the American Association for the Study of Liver Diseases (AASLD) and the British Association for the Study of the Liver (BASL) build upon this algorithm. Our patient scored 5 points on the EASL scale, which is sufficient for diagnosis. Nevertheless, we believe that she met the diagnostic criteria without having the actual disease. Newer guidelines by AASLD and BASL do not provide additional conclusivity. Our case demonstrates the need for re-evaluation of the diagnostic criteria of WD, where uncertainty can mean permanent hepatic and brain damage. Diagnostic guidelines should incorporate new biomarkers, ophthalmological techniques, and advanced technologies such as next-generation sequencing or CRISPR-Cas-based tools.

## Introduction

Wilson’s disease (WD), also known as hepatolenticular degeneration, is an autosomal recessive disorder of copper metabolism found worldwide [1,2]. The global prevalence of WD is estimated at 1/30,000 with a carrier rate of approximately 0.011 and gene frequency estimated at 0.56 [3,4]. There are over 300 disease-causing mutations of the *ATP7B* gene in addition to possible mutations of modifying genes [1,2,4-8]. As a result, genotype-phenotype relationships remain unknown [1,2,4-8]. Considering the heterogeneity in clinical presentation and the difficulties in diagnosing the condition, true prevalence may be much higher [3].

The pathogenesis of the disease involves mutations in the *ATP7B* gene, leading to impaired copper excretion. Accumulated copper produces reactive oxygen species that damage tissue in several organs, resulting in varied presentations. Frequently involved organs include the liver, brain, cornea, bones, and kidneys [1,2,4,5].

Liver damage is a hallmark feature of WD, as the defective ATP7B protein is expressed on hepatocytes [1,2]. The extent of liver involvement can range from asymptomatic lab abnormalities to cirrhosis or fulminant acute liver failure [2,9-12]. Symptoms can include jaundice, malaise, and nonspecific abdominal complaints, which are difficult to distinguish from symptoms of more common liver diseases, such as chronic or acute hepatitis [2]. A high degree of clinical suspicion is vital, as early diagnosis and rapid intervention can prevent irreversible liver damage and may also facilitate screening of at-risk family members [9].

As with hepatic manifestations, there is heterogeneity of neuropsychiatric complaints [2,7]. Neuromotor symptoms fall into three separate categories, namely, ataxic syndromes, akinetic rigidity characteristic of Parkinsonism, and a dystonic syndrome that can progress to disabling contractures. Presentations often include elements of each syndrome. Tremors classically manifest as an irregular, dystonic, proximal tremor with a wing-beating or flapping appearance but can also be a rest, action, or intention tremor [2,12]. Other characteristic manifestations include dysarthria, drooling, facial grimacing, and lip retraction [2,12]. At advanced stages of disease, structural damage may be detectable by brain MRI or CT [8]. A characteristic “face of the giant panda” sign can be seen on MRI in a minority of patients [2]. Behavioral and psychiatric symptoms precede hepatic or neurological symptoms in up to 30% of patients [2,7]. In children, neuropsychiatric symptoms may present as behavioral issues or declining academic performance, which may be misattributed to puberty [2,7]. Adult psychiatric manifestations can include behavioral and personality changes, anxiety, depression, and, less commonly, psychosis resembling schizophrenia [2,7,12].

Ocular disease most commonly presents as Kayser-Fleischer (KF) rings [2]. These are red, golden brown, or blue-green rings overlying the iris that occur due to copper deposits in Descemet’s membrane [2,11]. KF rings are a hallmark feature of WD, and they can be seen on slit-lamp examination in 90-100% of WD patients with neurological symptoms and half of patients with hepatic involvement. However, they are not specific for WD, and similar rings have been reported in other chronic cholestatic diseases, obstructive liver diseases, chalcosis, multiple myeloma, and pulmonary carcinoma [2,3,13-15]. Other, more rare ophthalmic presentations include sunflower cataracts, which are caused by copper accumulation at the center of the lens [2,15,16].

Diagnosis of WD relies on multiple methods, including clinical presentation, laboratory tests, imaging, and genetic analysis [1,2]. Globally, there are many diagnostic approaches, many of which are adaptations of the Leipzig scoring algorithm developed by the European Association for the Study of the Liver (EASL) in 2001 [2,12,17,18]. The Leipzig scoring algorithm attributes points for the presence or absence of KF rings (2 points), level of serum ceruloplasmin (up to 2 points), 24-hour urinary copper level (up to 2 points), Coombs-negative hemolytic anemia (1 point), genetic mutation analysis (up to 4 points), liver biopsy findings (1 point), and presence of neurologic symptoms (2 points) [2]. A score of 4 or more establishes a diagnosis, but lower scores do not rule out the disease. A score of 3 suggests the need for further testing, while a score of 2 or less indicates a low likelihood of WD.

In the United States, the most updated algorithm comes from the American Association for the Study of Liver Diseases (AASLD), which published practice guidelines in 2022 that build upon the EASL guidelines [15]. The AASLD proposes four diagnostic flowcharts based on the primary organ system involved (hepatic or neurologic) and the presence or absence of KF rings. Each flowchart then uses ceruloplasmin and 24-hour copper excretion levels to determine the likelihood of WD and whether further testing is necessary [15]. Internationally, the most recent guidelines were published in 2022 by the British Association for the Study of the Liver (BASL) [18]. According to the BASL, standard workup in suspected WD begins with complete blood count, transaminases, coagulation profiles, and serum ceruloplasmin measurements [18]. In those with unexplained liver disease, concomitant movement disorders, or other suspicious findings, wider screening should be done, including a 24-hour urinary copper excretion and slit-lamp examination. If the diagnosis is still uncertain, serum copper levels, genetic testing, liver imaging, liver biopsy, and neuroimaging may be indicated [18]. Of note, diagnosis frequently requires synthesis of several of these tests along with additional selected tests, which can include aspartate aminotransferase (AST)/alanine aminotransferase (ALT) ratio, alkaline phosphatase/total bilirubin ratio, and copper-65 absorption tests [2,18,19]. Yet, no single test or imaging modality confers a diagnosis or adequately rules out the disease [2,20,21]. Liver biopsy with elevated dry weight parenchymal copper content provides the best biochemical evidence of WD, but it is also present in some cholestatic diseases [2]. Additionally, levels can be underestimated due to the heterogeneous distribution of copper accumulation within the liver [2]. It is estimated that 18% of adults with diagnosed WD will not meet the threshold of 4 µmol/g dry weight hepatic copper content [2]. The BASL guidelines thereby summarize general directions for providers, but do not include specific threshold values or a clear diagnostic algorithm.

Genetic testing is similarly difficult, with over 600 possible mutations contributing to disease [2]. Allele-specific tests are used to assess for more common variants. However, such variants remain rare, and many patients are heterozygous for any one mutation but carry more than one mutation [2]. Identification of two or more mutations confirms the diagnosis [2].

Despite the many published algorithms, the marked variability in signs, symptoms, gene mutations, and involved organ systems poses challenges to clinicians attempting to definitively diagnose WD [2,3]. Currently, no one algorithm appears to comprehensively address the variability in disease signs and symptoms seen in this disease. Yet, timely diagnosis is critical in a population where delays can result in irreversible tissue damage in the brain, liver, and eyes [2,9]. In this article, we explore a case where current diagnostic guidelines were insufficient to rule in or rule out a diagnosis of WD. This case highlights inadequacies in the current guidelines and reveals an opportunity for their re-evaluation in a disease where diagnostic delays and inaccuracy can be severely detrimental to patients.

## Case presentation

A 54-year-old woman with a complex past medical history, including hepatitis C (treated with ledipasvir/sofosbuvir), cirrhosis, and hepatic encephalopathy, presented to an outside hospital with two months of lower back pain, fever, chills, cough, and progressive urinary incontinence. She had an extensive but nonspecific psychiatric and substance use history that was notable for polysubstance abuse (remote history of intravenous (IV) drug use, chronic opiate use, alcohol use disorder, tobacco dependence, and recent cocaine use) and longstanding affective and behavioral instability. She had had multiple psychiatric visits and hospitalizations since age 13, as well as multiple medication trials with limited benefit.

On transfer to our hospital for further evaluation, the patient exhibited tangential thought processes, flight of ideas, occasional clang associations, magical thinking, and unusual perceptual experiences. She also expressed paranoid or delusional thoughts against the medical staff about being abducted, having tests performed on her, and being fed poisoned hospital food. Collateral information from the patient’s sister and outside hospital records described a history of childhood abuse, unstable interpersonal relationships, and impulsivity, including binge eating and substance abuse. Prior records also noted a history of chronic dementia with superimposed acute mental decline that was demonstrated with poor performance on formal cognitive testing. Psychiatry consultation identified possible bipolar disorder and borderline personality disorder versus schizotypal personality disorder.

Continued investigation revealed a family history significant for early cirrhosis and a heterogeneous range of neuropsychiatric disorders with early cognitive decline, including a maternal aunt with bipolar disorder versus schizoaffective disorder and a brother who died of unspecified complications related to alcohol abuse.

Spinal imaging confirmed osteomyelitis of L3 to S1 with a noncompressive L3 epidural abscess, and blood cultures grew methicillin-resistant *Staphylococcus aureus *(MRSA). Abdominal ultrasound revealed a new mass on the right hepatic lobe.

**Figure 1:**
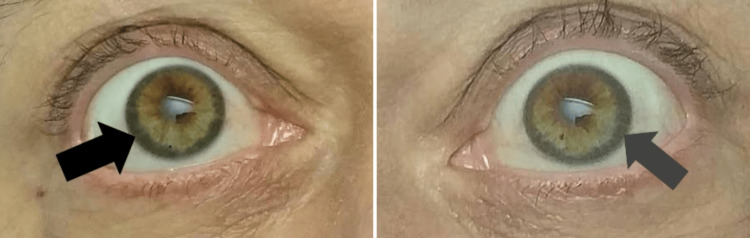
Symmetric, thick, dark greyish-blue deposits visible around the periphery of the patient’s corneas bilaterally, suggestive of Kayser-Fleischer rings and possible copper deposition within the Descemet’s membrane.

On ophthalmologic examination, greyish-blue rings were noted around both eyes, consistent with KF rings (Figure 1). Considering the strong indicator of these ocular manifestations as signs of copper accumulation, along with the patient’s multiple hepatic and neuropsychiatric symptoms, it was considered that she may have undiagnosed WD.

The standard workup for this diagnosis was begun, including levels of ceruloplasmin and 24-hour urinary copper excretion. The patient’s modestly low ceruloplasmin of 0.176 g/L (reference range >0.2 g/L) was consistent with expected values in WD. Nevertheless, 24-hour urinary copper excretion was well within normal limits with a value of 8 μg over 24 hours (reference range = 3-40 μg/24 hours).

The differential diagnosis for this patient included, but was not limited to, hepatic encephalopathy secondary to cirrhosis, metabolic or toxic encephalopathy related to substance use, and WD presenting with neuropsychiatric, ocular, and hepatic involvement.

The patient received zinc, lactulose, rifaximin, psychiatric medications, and a prolonged course of IV vancomycin and daptomycin. Her condition gradually improved, and after an extended inpatient stay, she was discharged to a long-term acute care facility for continued antibiotic therapy. Despite demonstrating some features consistent with WD, there were alternative explanations for her clinical findings that were much more likely than WD. Given the highly invasive nature of liver biopsy in an already ill patient, more common explanations for her condition, and her clinical improvement, we did not conduct further diagnostics during her recovery in our hospital, nor did we diagnose her with WD, even though she exhibited several findings suspicious for the disease. The patient was lost to follow-up, and further diagnostics were not obtainable. The patient provided consent for the publication of her data and images.

## Discussion

Diagnostic challenges

Our patient scored a total of 5 points on the Leipzig scoring algorithm for the presence of KF rings (2 points), severe neurologic symptoms (2 points), and serum ceruloplasmin between 0.1 to 0.2 g/L (1 point). Therefore, she exceeded the threshold value of 4 points for establishing a diagnosis of WD (Table 1). Nevertheless, considering our patient’s multiple comorbidities, there is a strong possibility that she met the criteria without actually having WD [2,20,21]. It is possible that her liver disease resulted from cumulative damage due to disordered copper metabolism. However, in this case, her hepatic disease was likely a result of chronic alcohol use and hepatitis C. Her complicated psychiatric history may have been the result of copper accumulation in the brain, but could just as easily be attributed to an inherent personality disorder complicated by polysubstance use, hepatic encephalopathy, history of abuse as a child, or altered mental status secondary to MRSA bacteremia.

**Table 1:** Summary of clinical signs in our case with scoring according to 2001 Leipzig guidelines. Our patient had a score of 5, sufficient for a diagnosis of WD, despite a high level of diagnostic uncertainty and confounding conditions in this complex case.

Criteria or clinical sign	Value	Leipzig reference range and corresponding score	Leipzig criteria score
Kayser-Fleischer rings	Present	Present (2) Absent (0)	2
Neurological symptoms	Severe	Severe (2) Mild (1) Absent (0)	2
Serum ceruloplasmin (g/L)	0.176	<0.1 (2) 0.1–0.2 (1) Normal >0.2 (0)	1
24-hour urinary copper excretion (µg/24 hours)	8	>2× upper limit of normal (2) 1–2× upper limit of normal (1) Normal, 3–40 (0)	0
Coombs-negative hemolytic anemia	Absent	Present (1) Absent (0)	0
Total liver copper (µmol/g)	Not assessed	>5× upper limit of normal (2) Increased, 0.8–4 (1) Normal, <0.8 (-1) rhodanine-positive granules present (1)	Not assessed
Genetic mutation	Not assessed	Present on both chromosomes (4) Present on 1 chromosome (1) Absent (0)	Not assessed
Total score		≥4 Diagnosis established 3 Diagnosis possible ≤2 Diagnosis unlikely	5

**Figure 2:**
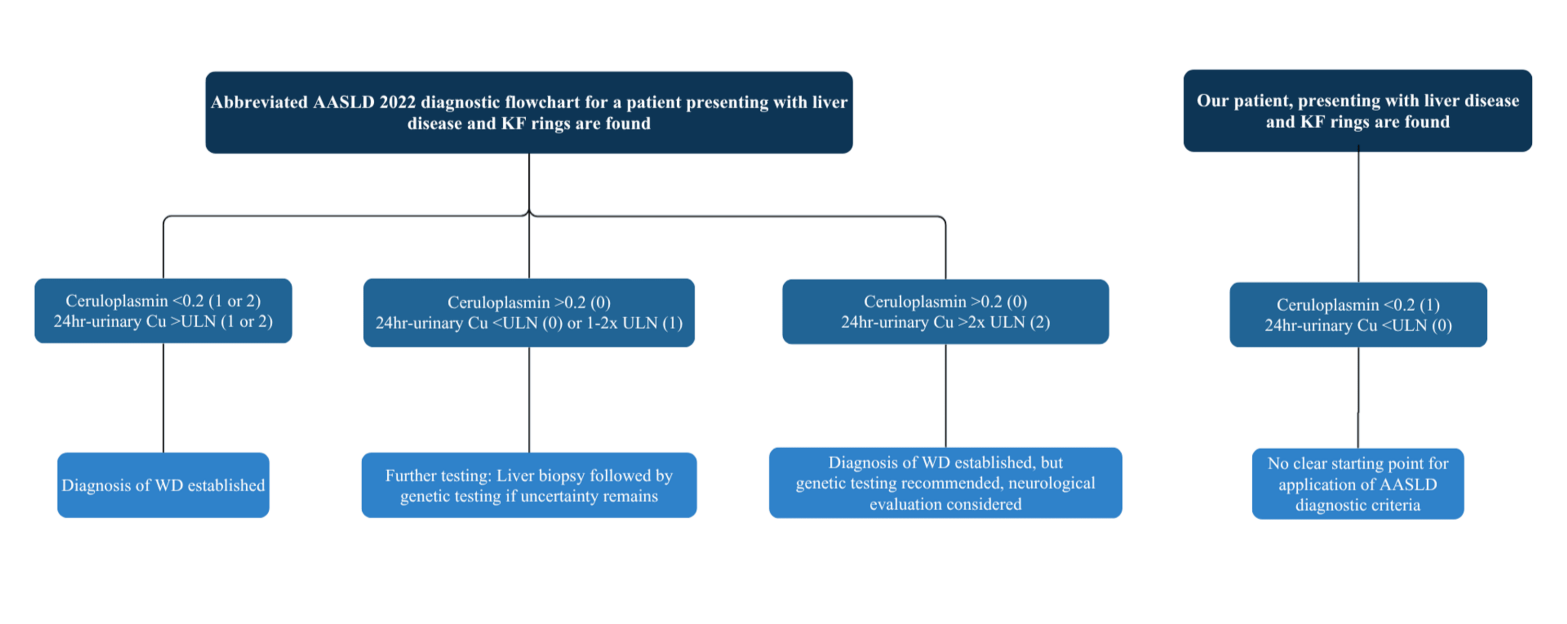
The flowchart to the left represents an abbreviated version of the American Associated for the Study of Liver Diseases (AASLD) 2022 Wilson’s disease (WD) diagnostic algorithm for a patient who presents with liver disease and the presence of Kayser-Fleischer (KF) rings. The right flowchart presents lab values from our patient, demonstrating that they do not fit clearly into any of the three AASLD pathways for diagnosis and underscoring both the inapplicability of these guidelines and the continued diagnostic uncertainty in our patient’s case. Ceruloplasmin is reported in units of g/L. 24-hour urinary copper excretion (24-hr urinary Cu) is reported relative to the upper limit of normal values (ULN) of 40 μg/24 hours. Values in parentheses refer to the corresponding score within the Leipzig 2001 diagnostic algorithm.

Utilizing the 2022 BASL and AASLD criteria does not provide additional clarity. BASL guidelines recommend using clinical judgement to interpret a combination of laboratory tests. Based on their suggestions, we believe that her comorbidities better explain her Wilson-like presentation [18]. Meanwhile, the AASLD flowcharts are difficult to interpret in our patient’s case, as there is no diagnostic flowchart pathway for a patient with both low ceruloplasmin and normal 24-hour urinary copper excretion (Figure 2) [15].

One challenge in diagnosing WD lies in the symptomatic overlap with more common liver and brain conditions. Hepatic encephalopathy, for example, is much more commonly diagnosed in patients with concomitant hepatic and neurological symptoms, and the two disorders can be difficult to distinguish using symptoms alone [22]. The tremor seen in WD can mimic hepatic asterixis, and both conditions can impact basal ganglia function [12,22]. Indeed, it is possible that in patients with WD, hepatic encephalopathy may be a contributor to neuropsychiatric symptoms alongside copper toxicity. No single test can reliably differentiate the two conditions [22].

Simultaneously, it is well established that neuropsychiatric conditions and substance use disorders are frequently comorbid [23]. Patients with known neuropsychiatric conditions therefore carry an increased risk for developing liver damage related to substance use, including hepatitis and cirrhosis [20]. Our case demonstrates the complexity in teasing apart these symptoms in the diagnosis of WD.

Even after WD is suspected, the lab values designed to help distinguish it from similarly presenting conditions, including serum ceruloplasmin levels and urinary copper excretion, are not sensitive or specific enough to provide entirely definitive data [2,20,21]. One systematic review suggests that for serum ceruloplasmin, a threshold value of <0.20 g/L (0.176 g/L in our patient) has a sensitivity of 77.1-99.0% and a specificity of 55.9-82.8% [24]. The low specificity results in difficulty ruling out other conditions, such as malabsorption or severe liver disease, in the setting of this patient’s chronic hepatitis, cirrhosis, and alcohol use disorder [25]. For 24-hour urinary copper excretion, a cut-off value of 40-100 μg/24 hours (8 μg/24 hours in our patient) carries a sensitivity of 50-80% and a specificity of 75.6-98.3% [24]. While 24-hour urinary copper excretion can be normal in 16-23% of diagnosed WD cases, this is more often the case in pediatric patients who are earlier in the disease course or asymptomatic individuals [24,25]. The AASLD suggests that normal 24-hour urinary copper excretion in combination with a low serum ceruloplasmin is highly atypical in symptomatic WD [15]. However, the presence of KF rings is considered to have a high specificity for WD in a suspicious clinical context [26]. Despite meeting the Leipzig criteria for WD diagnosis, our patient’s laboratory findings together rule down her likelihood of WD. Yet, in the context of KF rings, it cannot be ruled out.

Other tests, such as liver biopsy and measurement of hepatic parenchymal copper, are highly invasive and can be unreliable due to uneven distribution of copper in hepatic tissue [2,3]. Finally, neurological findings remain unclear. While neurologic scales have been suggested to quantify symptoms, there is no consensus on the scoring of neuropsychiatric symptoms [15,25]. Brain MRI findings may include basal ganglia lesions or a characteristic “face of the giant panda” sign, but thalamic and brainstem lesions are also regularly seen, and lesions are frequently absent in those without gross neuropsychiatric symptoms [2]. In a condition such as WD, which can cause irreversible hepatic and neurological damage if left untreated, this level of diagnostic ambiguity is unacceptable.

Just as it is important for clinicians to possess the ability to diagnose WD, it is equally important to be able to rule it out. Treatment for WD is lifelong and emphasizes zinc salts to decrease intestinal absorption and copper chelators to reduce copper stores [2,3,12]. In patients with acute liver failure, a liver transplant can be necessary [2,3]. Should patients be wrongly diagnosed with WD, as our patient seemed to be, taking such measures can be dangerous for drug recipients, depleting the body of necessary copper stores and provoking anemia, nausea, and neurological issues [27].

New diagnostic methods

Current diagnostic guidelines are not up to date with the advances in knowledge and growing awareness of what was once believed to be a rare disease. While the Leipzig scoring algorithm and its successors have utility for identifying phenotypic features that suggest WD, advances in technology have made it easier and more affordable to confirm the diagnosis. Several new diagnostic tools have emerged as potentially useful options for clinicians. The exchangeable copper (CuEXC) assay biomarker allows for calculation of a ratio between CuEXC (which is postulated to represent copper bound to albumin) and total serum copper, providing information on labile or free serum copper. This diagnostic shows promise, with higher sensitivity and specificity for WD diagnosis [28]. Such tests are becoming increasingly cheaper, faster, and more accurate.

Ophthalmologic detection of KF rings, classically found via slit-lamp, may be enhanced by alternative tools such as Scheimpflug imaging and anterior-segment optical coherence tomography, which, in conjunction with slit-lamp examination, may allow for improved quantification of these KF rings [29,30].

Next-generation sequencing

Current innovations now allow for speedy and accurate diagnosis, namely, next-generation sequencing. This might be applied for rapid genetic analysis of the 21 exons coding *ATP7B*. It may be especially useful for diagnosing WD in the setting of acute liver failure, where biomarkers such as urinary copper have little utility [31]. One study in France found that 98% of patients with known WD have at least two mutations to the *ATP7B* gene [21]. With next-generation sequencing, molecular analysis has become more accessible and may act as a more rapid confirmatory test, initiating proper treatment.

There is growing discussion of an epigenetic component to WD, which may help explain differing phenotypes, even among those with the same disease-causing mutation [32]. A number of modifier genes have been proposed as contributors to WD [19,32]. Next-generation sequencing methods, such as bisulfite conversion, are used to measure DNA methylation and may be a path toward identifying epigenetic modification to the culprit *ATP7B* and modifier genes [19]. Such studies could elucidate a clearer genotype-phenotype relationship in WD. They may also aid in screening at-risk family members and determining the need for aggressive treatment measures.

CRISPR-Cas diagnostics

CRISPR-Cas diagnostics is also suggested as a quick and reliable diagnostic tool for WD [33]. While its use is limited by the large number of *ATP7B* mutation variants, its portability and relative cost-effectiveness confer access advantages, and it may become useful in testing for more common gene variants [33].

## Conclusions

At present, our diagnostic toolbox for WD lacks certainty. As new laboratory tests and technological advances surface and become more readily available, we should investigate their practicality for incorporation into clinical practice. Future WD diagnosis should consider more heavier reliance upon such molecular tests to expedite diagnosis and improve patient outcomes. Given the importance of decisive and early diagnosis on clinical patient outcomes, our case demonstrates the need for critical re-evaluation of the WD diagnostic criteria.
